# In vitro biological effects of two anti-diabetic medicinal plants used in Benin as folk medicine

**DOI:** 10.1186/1472-6882-13-51

**Published:** 2013-03-01

**Authors:** Fifa TD Bothon, Eric Debiton, Felicien Avlessi, Christiane Forestier, Jean-Claude Teulade, Dominique KC Sohounhloue

**Affiliations:** 1 Clermont University, Université d’Auvergne, IMTV, F-63000, Clermont-Ferrand, France; 2 INSERM, UMR 990, IMTV, F-63005, Clermont-Ferrand, France; 3 University of Abomey Calavi, Laboratoire d’étude et de Recherche en chimie Appliquée, Ecole Polytechnique d’Abomey Calavi, Cotonou, Benin; 4 Clermont University, Université d’Auvergne, Laboratoire Microorganismes: Génome et Environnement (LMGE), BP 10448, F-63000, Clermont-Ferrand, France; 5 CNRS, UMR 6023, LMGE, F-63177, Aubiere, France

**Keywords:** *Polygonum senegalensis*, *Pseudocedrela kotschyi*, α-glucosidase, Antioxidant, Antibacterial

## Abstract

**Background:**

Extracts from *Polygonum senegalensis* (Polygonaceae) and *Pseudocedrela kotschyi* (Meliaceae) are two important traditionally used medicinal plants in rural Benin to treat many diseases and notably type 2 diabetes. The aim of the study was to investigate the α-glucosidase inhibition, antioxidant and antibacterial activities of those plants extract: *Polygonum senegalensis* leaves, and *Pseudocedrela kotschyi* root.

**Methods:**

Hydro-alcoholic (50%) extracts were analyzed for their phytochemical content and tested for their inhibition potency on α-glucosidase from *Saccharomyces cerevisiae*. Antioxidant activities were assessed using the DPPH, ORAC, FRAP and DCFH-DA (cell based) assay. Finally, the antibacterial activity was evaluated using MIC determination on four Gram-positive *cocci* (*Bacillus subtilis*, *Clostridium difficile*, *Enterococcus faecalis, Staphylococcus aureus*), three Gram-negative *bacilli* (*Escherichia coli*, *Pseudomonas aeruginosa*, *Klebsiella pneumoniae*), and the yeast *Candida albicans*.

**Results:**

Each extract presented significant α-glucosidase inhibition and antioxidant activities. *Polygonum senegalensis* leaf extracts were the most active in each *in vitro* assay with an IC_50_ = 1.5 μg/ml for α-glucosidase inhibition and an IC_50_ = 6.8 μg/ml for DPPH scavenging, - 4.5 μmol Fe II/g of dry matter - 9366 μmol Trolox / g DW - for FRAP and ORAC values, respectively. IC_50_ = 2.3 μg GA / ml for DCFH-DA assay. Concerning its antibacterial activity, a growth inhibitory effect was observed only against three Gram negative bacilli: *B. subtilis, E. faecalis, S. aureus* and the yeast *C. albicans* at high concentration.

**Conclusion:**

The results showed that the semi alcoholic extract of the two studied plants possess α-glucosidase inhibitory activity, antioxidant potency, and low antibacterial effect.

## Background

Free radicals and other reactive oxygen species (ROS) generated in living organisms participate in many diseases including cancer, cardiovascular diseases, immunodeficiency, liver injury and infections [1]. Due to the huge costs of modern therapy for developing countries, the World Health Organization has estimated that 80% of the world’s population use botanical medicine for their primary health care needs; so alternative strategies are urgently needed [2]. Many Plants were used in traditional medicine to treat a lot of pathologies. They play an important role in the life of rural people, particularly in remote parts of developing countries which do not possess important health facilities. *Polygonum senegalensis* (polygonaceae) and *Pseudocedrela kotschyi* (meliaceae) are two important traditional medicinal plants used in rural Benin to treat many diseases. They are used to treat diabetes mellitus, hypertension, gynaecological and wound infections. Some of these properties have been confirmed in other studies. For example *Pseudocedrela kotschyi*’s antidiabetic [3] and antibacterial activities [4] were previously described. However, no mechanisms of action were described.

Recent interest in plant polyphenols has focused on their potential benefits to human health. The polyphenols are capable not only to reduce oxidative stress but also to inhibit carbohydrate hydrolyzing enzymes and thus preventing hyperglycemia [5,6]. Therefore, this class of compounds generally possesses high antioxidant and antidiabetes potencies. However, systematic screening of common plants to correlate the relationships of their polyphenolic contents with their biological activities has yet to be investigated in edible plants from Benin.

The aim of the present work was to study and to evaluate the biological activities of both plants as an α-glucosidase inhibitor, an antioxidant, and an antibacterial.

## Methods

### Chemicals

α-Glucosidase from *Saccharomyces cerevisiae* EC.3.2.1.20, 4-Nitrophenyl α-D-glucopyranoside, Acarbose, L-Ascorbic acid, 2,4,6-Tris(2-pyridyl)-s-triazine (TPTZ); 2,2^′^-Azobis(2-methyl-propionamidine) dihydrochloride; Fluorescein Sodium Salt, Folin-Denis’ reagent, 2,2-diphenyl-1-picrylhydrazyl, Gallic acid, Quercetin, Vanillin, (+)-Catechin hydrate, Iron(II) sulfate heptahydrate, were purchased from Sigma (St Quentin Fallavier, France). Iron (III) chloride hexahydrate and Sodium carbonate were purchased from Prolabo (Fontenay-sous-Bois, France). Phosphate buffered saline was purchased from Gibco, and anhydrous Aluminum chloride was purchased from Acros Organics (Halluin, France).

### Microorganisms

*Bacillus subtilis* (ATCC 633), *Clostridium difficile* (ATCC 9689), *Enterococcus faecalis* (ATCC 29212), *Staphylococcus aureus* (ATCC 25923), *Escherichia coli* (ATCC 8739), *Pseudomonas aeruginosa* (ATCC 27853), *Klebsiella pneumoniae* LM21 (Favre-Bonte, 1999) and *Candida albicans* (ATCC 10231) were kindly provided by the LMGE. All these strains were kept frozen at −80°C in appropriate media with 15% of glycerol.

### Collection of plant materials and preparation of extracts

Different parts of the studied plants were chosen in the light of a bibliographic survey on traditional medicinal plants. Identification of the plants was done in the field by a botanist from the National Herbarium of Bénin (HNB). *Polygonum senegalensis* leaves harvested in February 2010 in Adjohoun and the roots of *Pseudocedrela kotschyi* (Schweinf) in September 2009 in Gbegourou-Binassi in the north of Bénin. These species, which belong to the families Polygonaceae, and Meliaceae respectively, were identified and a voucher was kept at the HNB under the respective numbers: AA6384/HNB and AA6389/HNB.

One hundred g of plant powder was poured into 1000 ml of hydroalcoholic solution (50% v/v), the whole mixture was then mechanically stirred for 3 h, at 25°C. The solution was left for 1 h to settle. It was then filtered with Whatman N°1 paper using a Buchner and a vacuum pump. The filtrates were collected and evaporated in a rotary evaporator at 40°C. The crude extracts obtained were stored at −18°C until use.

### Phytochemical analysis

#### Preliminary qualitative phytochemical screening

For each extract, colorimetric tests were used to reveal the families the compounds belong to, such as the foam index technique, the Shibata test and Fehling’s test which respectively identifies Saponins, flavonoids and reducing compounds [7]. Tannins were revealed using FeCl_3_ and Stiasny’s reagent according to Soro *et al.*[8] and Dragendorff’s test for alkaloids [9].

#### Determination of total phenolic content

Total phenolic contents were determined using Folin-Denis’ reagent following a slightly modified method of Ferreira *et al.*[10]. A mixture of 10 μl of plant extract, 790 μl of distilled water and 50 μl of Folin reagent was vigorously homogenized. After 5 min, 150 μl of sodium carbonate 7.5% (w/v) were added and the reaction mixture was incubated at room temperature for 30 min. The absorbance was then measured at 760 nm using the UV–vis spectrophotometer 1800 Shimadzu (Kyoto, Japan). Gallic acid was used as a reference and for the calibration curve; results were expressed in gram of gallic acid equivalent per g of dry weight (DW).

#### Total flavonoid content

The total flavonoid content was determined according to Quettier-Deleu *et al.* using AlCl_3_[11]. This method is based on the formation of a flavonoid-aluminum complex with maximum absorbance at 430 nm: 500 μl of extract was added to the solution of AlCl_3_ (2% (m/v) in methanol) following incubation for 15 min at room temperature, the absorbance of the mixture was measured at 430 nm with a UV–vis spectrophotometer type Shimadzu UV–vis 1800. The flavonoid content was calculated from a quercetin standard curve and expressed in mg quercetin equivalent per g of DW.

#### Condensed tannins content

Condensed tannins content was determined according to Price *et al.*[12]. To 200 μl of extract, 1 ml of methanolic solution of vanillin [mixture of equal volumes of 8% (v/v) HCl at 37% in methanol and 4% (m/v) vanillin in methanol] was added. The mixture was kept at 30°C for 20 min and the absorbance read at 500 nm using a spectrophotometer Shimadzu UV–vis 1800. The tannins content was calculated from catechin standard curves, and expressed in mg catechin per g of DW.

### Biological effects

#### α- Glucosidase inhibitory activity

The slightly modified method of Rao *et al.*[13] was adopted to determine the α-glucosidase’s inhibitory activity. Briefly, in a 96-well microplate 100 μl of a sample of different concentrations was incubated with 50 μl α-glucosidase (1.0 U/ml) (from *Saccharomyces cerevisiae*) in phosphate buffer (0.1 M, pH 6.8) for 10 min at 37°C. The reaction was initiated by addition of 50 μl of substrate: 5 mM, *p*-nitrophenyl-α-D glucopyranoside in a 0.1 M phosphate buffer at pH 6.8. P-nitrophenol’s release kinetics were measured spectrophotometrically with a microplate spectrophotometric reader Multiskan MS™ (Labsystems, Minneapolis, USA) for 5 min with intervals of 30 seconds at 405 nm. Acarbose was used as reference. The IC_50_ (concentration required to decrease the reaction rate to 50%) was then determined from the concentration-dependence curve.

#### Antioxidant activity

##### 2, 2-diphenyl-1-picrylhydrazil (DPPH) radical scavenging assay

Free radical scavenging activity was determined using 2,2-diphenyl-1-picrylhydrazil (DPPH), as described by Povichit *et al.* with slight modifications [14]. Briefly, in a 96 well microplate, 180 μl of DPPH solution (6.10^-5^ M in Methanol) and 20 μl of the plant extract at different concentrations were added. The reaction mixture was shaken and incubated in the dark for 30 min at 37°C. The absorbance was read at 540 nm against blank using the microplate spectrophotometric reader. Ascorbic acid was used as positive control. DPPH radical scavenging activity was calculated according to the formula:

$$\left[\left(\mathrm{Abs}.\;\mathrm{of}\;\mathrm{control}\;-\;\mathrm{Abs}.\;\mathrm{of}\;\mathrm{sample}\right)/\mathrm{Abs}.\;\mathrm{of}\;\mathrm{control}\right]*100$$

And the IC_50_ value for each sample was calculated from the non-linear regression curve.

##### Ferric reducing/antioxidant power (FRAP) assay

The ferric reducing property of the extract was determined by using the assay described by Piljac-Zegarac *et al.*[15] and Mukherjee *et al.*[16] with slight modifications. Briefly 180 μl of a freshly prepared solution of FRAP reagent (mixture solutions: 300 mM acetate buffer, pH 3.6, 10 mM TPTZ in HCl 40 mM and FeCl_3_.6H_2_O 20 mM at a ratio of 10: 1: 1 at 37°C) are distributed in each well of a plate with 20 μl of sample. After briefly vortexing and 4 min of incubation in the dark, the absorbance of the colored product (ferrous tripyridyl triazine complex) was read at 595 nm using the microplate spectrophotometric reader. The standard curve was linear between 16 and 250 μM FeSO_4_.7H_2_O. Results were expressed in μmol Fe II per g of DW.

##### Oxygen Radical Absorbance Capacity (ORAC) assay

ORAC assay was carried out as described by Ou *et al.*[17]. The reaction was performed at 37°C with a microplate fluorescent reader Fluoroskan Ascent FL™ (Labsystems, Minneapolis, USA) at an excitation wavelength of 485 nm and an emission wavelength of 530 nm. In each well, 50 μl of fluorescein, (78 nM in PBS 75 mM, pH 7) and 50 μl of sample, PBS (blank) or trolox (standard) were added. The plate was then incubated at 37°C for 10 min. The oxidation reaction was then initiated by adding 25 μl of 2, 2-azobis-(2-amidinopropane) dihydrochloride (AAPH, 221 mM). Finally the absorbance was read at t = 0 and every 5 min until the fluorescence intensity became less than 5% of its initial reading value.

ORAC values are expressed as μmol Trolox equivalents per gram of dry matter, through the calculation of net AUC. Net AUC is the net area under the fluorescein decay curve.

$$\mathrm{AUC}=\left(1+f_{5}/f_{0}+f_{10}/f_{0}+\ldots F_{n+5}/f_{0}\right)*5$$

$$\begin{array}{cc} f_{0}=\mathrm{initial}\;\mathrm{fluorescence} & f_{n}{}_{+5}=\mathrm{fluorescence}\;\mathrm{at}\;\mathrm{time}\;\left(\mathrm{in}\;\min\right) \end{array}$$

##### Dichlorofluorescin diacetate ( DCFH-DA Cell-based assay )

Antioxidant activities on cells were evaluated using the compound 2^′^, 7^′^-Dichlorofluorescein diacetate (DCFH-DA) probes [18].

L-929 cells were cultured in Minimum Essential Medium with Glutamax® (Gibco-BRL) complemented with 10% (v/v) fetal calf serum (Gibco-BRL), Gentamicine (Gibco-BRL) 4 μg/ml, sodium pyruvate 10 mM, vitamins and free amino acids mixture (Gibco-BRL). For each experiment, cells were plated in black flat clear bottom 96-well microplates at 5x10^4^ cells per well and incubated for 24 h at 37 C and 5% CO_2_. After elimination of the medium, the microplates were incubated for 30 min with 100 μl HBSS/Hepes (15 mM, pH 7.4) containing 20 μM DCFH-DA. The cells were then washed with 200 μl of HBSS/ Hepes. To assess antioxidant activity, the cells were incubated with increasing concentrations of extracts in the absence or presence of 200 μM tert-butylhydroperoxide (t-BuOOH). Fluorescence was measured immediately after t-BuOOH addition and every 30 min during 90 min on the microplate fluorescent reader using an excitation wavelength of 485 nm and an emission wavelength of 530 nm. Results are express as IC_50_ (concentration required to decrease the reaction rate to 50%) is then determined using the concentration-dependent curve.

### Antibacterial assays

The cells from the stock cultures were inoculated in Müller-Hinton Broth (MHB) and incubated at 37°C under stirring for 24 h. The suspensions were then diluted with fresh MHB to achieve optical density (620 nm) of 10^6^ colony forming units per ml (CFU/ml). Dilutions to get the final concentration ranging from 0.156 to 10 mg/ml of extract in MHB were prepared in a 96-well microtiter plates in a volume of 100 μl. Finally 100 μl of each microorganism suspension (10^6^ CFU/ml) were added. A negative control composed of 100 μl of microorganism suspension and 100 μl of MHB was also included. The Minimum Inhibitory Concentration (MIC) was determined as the lowest concentration of the test samples that inhibited the growth of the tested microorganisms by visual assessment.

### Statistical analysis

All experiments were performed at least in triplicate, and results are expressed as mean ± SEM. Statistical analysis was performed using the statistical software XLSTAT (version 2012. 1.01, Addinsoft, Paris, France). The results are analyzed by the univariate ANOVA test followed by Dunnett/Tukey test for multiple comparisons and determination of significance level. Group means were considered to be significantly different at P < 0.05.

## Results

### Phytochemical analysis

As shown in Table 1, the presence of flavonoids, tannins, saponins and reducing compounds seem to be present in the extracts in significant amount. Conversely, the alkaloids were rare. The spectrophotometric results indicated that all the extracts contained significant amounts of total polyphenolic compounds, flavonoids and condensed tannins (Table 2). There was a significant difference in the flavonoids and condensed tannins concentration in both extracts. A higher concentration of flavonoids was found in the extract of *P. senegalensis,* but the tannin content was almost the same.

**Table 1:** Total chemical composition of the studied extracts

**Chemical composition**	***P. kotschyi***	***P. senegalensis***
Flavonoids	+++	+++
Tannins	+++	+++
Alkaloids	**-**	+
Saponins	+++	+++
Reducing compounds	+++	+++

- = Negative result, + = Positive result, +++ = Very high concentration.

**Table 2:** Total phenolics, flavonoids and condensed tannins contents

**Extracts**	**Polyphenolics content**	**flavonoids content**	**Condensed tannins content**
	**mg GAE/g DW**	**mg QE/g DW**	**mg CE/g DW**
*P. kotschyi*	91 ± 2^b^	0.24 ± 0,01^b^	456 ± 105^a^
*P. senegalensis*	126 ± 8^a^	8 ± 1^a^	488 ± 52^a^

In each column, the means with different symbol superscript a or b showed a significant difference at P < 0.05.

### α- Glucosidase inhibitory activity

α-Glucosidase inhibitors (AGIs) were among the available glucose-lowering medications. The glucosidase enzyme is located in the brush border of the small intestine and is required for the breakdown of carbohydrates and absorption of monosaccharids. The AGIs delay, but do not prevent, the absorption of ingested carbohydrates, reducing the postprandial glucose and insulin peaks [19]. Testing the α-glucosidase inhibitory effect of those four plants, might contribute to the understanding of their mechanisms of action.

**Figure 1:**
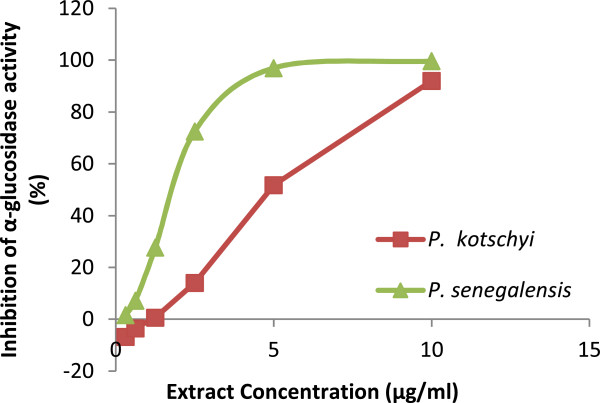
The α-glucosidase inhibitory activity of the extracts of the two plants at increasing concentrations.

The α-glucosidase inhibitory activity of the extracts of both plant species were compared on the basis of their IC_50_ values obtained. α- glucosidase inhibitory activity was observed with IC_50_ values of 1.50 ± 0.06 μg/ml in *P. senegalensis*, and 5.0 ± 0.2 μg/ml in *P. kotschyi*. Figure 1 showed that the extracts displayed strong α-glucosidase inhibitory activity in a dose dependent manner, with a significant difference in the inhibitory activity between both extracts (p < 0.0001). Moreover, each extract showed higher α-glucosidase inhibitory activity than the reference compound acarbose (IC_50_ 726 ± 15 μg/ml).

### Antioxidant activity

Several antioxidant assays are frequently used to estimate antioxidant capacities in fresh fruits and vegetables, these assays can roughly be classified into two types: assays based on hydrogen atom transfer (HAT) reactions and assays based on electron transfer (ET) [20]. In this work, we tested two types of ET assay: 2, 2- diphenyl-1-picrylhydrazyl (DPPH) and Ferric Reducing Antioxidant Power (FRAP) and one HAT assay: Oxygen Radical Absorption Capacity (ORAC).

**Figure 2:**
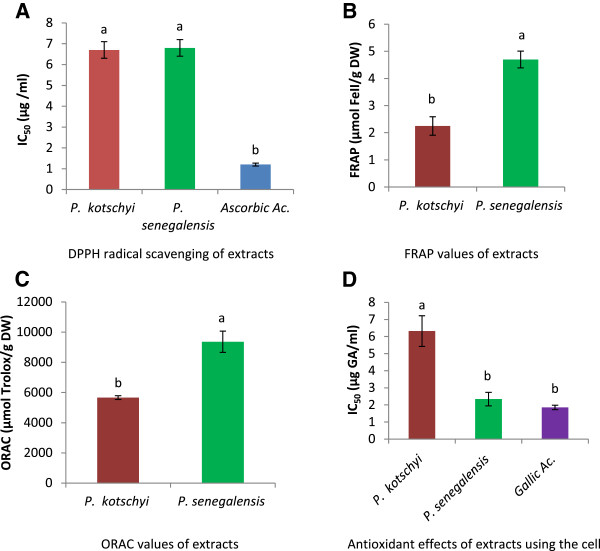
**Antioxidant activity of extracts. A**: 2, 2-diphenyl-1-picrylhydrazil (DPPH) radical scavenging, **B**: Ferric Reducing Antioxidant Power (FRAP) values, **C**: Oxygen Radical Absorbance Capacity (ORAC), **D**: Antioxidant effects of extracts using the DCFH-DA cell-based assay.

Antioxidant activity results are shown in Figure 2. DPPH radical scavenging activities of the different extracts are shown in Figure 2A by the value of their IC_50_: *P. kotschyi* (6.7 ± 0.4 μg/ml), *P. senegalensis* (6.8 ± 0.4 μg/ml) and the positive control L-ascorbic acid was 1.25 ± 0.07 μg/ml.

The FRAP assay measures the reducing potential of an antioxidant that reacts with a ferric tripyridyltriazine (Fe3+−TPTZ) complex to produce a colored ferrous tripyridyltriazine (Fe2+−TPTZ). The FRAP values of the two extracts were: 5.0 ± 0.3 μmol Fe II / g DW for *P. senegalensis* and (2.3 ± 0.3 μmol Fe II / g DW) for *P. kotschyi*. There is a statistically significant difference between the two extract (Figure 2B). ORAC assay is widely employed to determine antioxidant content of foods using fluorescein as a probe for oxidation by peroxyl radical. Figure 2C shows the ORAC values of the extracts.

Therefore, a one-dimensional assay protocol cannot be used alone to test all relevant parameters. A complementary method used to evaluate the antioxidant activities of fruit and vegetable extracts directly in living mammalian cells could be useful. Unfortunately, very few studies had used cell culture models to evaluate antioxidant potential. The intracellular reactive oxygen accumulation as reflected by the DCFH fluorescence was measured in this assay. DCFH-DA is a useful indicator of reactive oxygen species (ROS) and oxidative stress. The non-polar and non-ionic DCFH-DA cross cell membranes and are hydrolyzed by intracellular esterase to non-fluorescent 2^′^, 7^′^-dichlorofluorescin (DCFH). In the presence of ROS such as hydrogen peroxide (H_2_O_2_), lipid hydroperoxides or peroxinitrites, DCFH is oxidized to fluorescent 2^′^, 7^′^-dichlorofluorescein (DCF). Therefore, DCFH is useful to indirectly measure the effect of intracellular antioxidant activities in scavenging the ROS and in protecting the DCFH from the oxidation [20]. The value of IC_50_ was expressed in μg equivalent of gallic acid per ml of extract. The IC_50_ was calculated and demonstrated that gallic acid (1.8 μg / ml), *P. Senegalensis* (2.3 μg GA / ml), *P. Kotschyi* (6.3 μg GA / ml) extracts have good activity on ROS inhibition in cells (Figure 2D).

### Antibacterial activity

Table 3 shows the MICs of plant extracts against the 7 tested bacteria and the yeast. None of the plant extract was able to impair growth of all the 8 microorganisms tested and the growth inhibitions observed were strain related (Table 3). Indeed, *C. albicans* growth was inhibited by all plant extracts at concentration of 20 mg/ml. Among the tested bacteria, *B. subtilis*, *E. faecalis* and *S. aureus* growth were inhibited at concentration equal to 20 mg/ml or even lower with *P. senegalensis* extract for *E. faecalis*.

**Table 3:** Minimum inhibitory concentrations of studied extracts (mg/ml)

**Microorganism**		**Extract**	
		***P. senegalensis***	**P. kotschyi**
	*B. subtilis*	**5**	**10**
**Gram-positive**	*C. difficile*	**ND**	**ND**
	*E. faecalis*	**1,25**	**20**
	S. aureus	**5**	**5**
	*E. coli*	**ND**	**ND**
**Gram-negative**	*K. pneumoniae*	**ND**	**ND**
	*P. aeruginosa*	**ND**	**ND**
**Yeast**	*C. albicans*	20	**20**

ND = not detected.

## Discussion

The aim of the present study was to establish the potential α-glucosidase, antioxidant and antimicrobial activities of hydro-alcoholic extract of two plants used in traditional medicine in Benin: *Polygonum senegalensis*, and *Pseudocedrela kotschyi*.

Type 2 diabetes is an endocrine disease, which accounts for 9% of deaths worldwide. The aim of oral therapy is to reach normoglycemia to prevent later complications. Among glucose-lowering medications, α-glucosidase inhibitors which delay the absorption of ingested carbohydrates, reduces the postprandial glucose and insulin peaks [21]. Acarbose represents a pharmacological approach to achieving the metabolic benefits of a slower carbohydrate absorption in diabetes, by acting as a potent competitive inhibitor of intestinal α-glucosidases. Acarbose molecules attach to the carbohydrate binding sites of α-glucosidases, with a much higher affinity constant than the normal substrate [21]. However, the conversion of oligosaccharides to monosaccharides is only delayed rather than completely blocked due to the reversible nature of the inhibitor-enzyme interactions.

In our study acarbose was used as the positive control; it inhibited the α-glucosidase activity with an IC_50_ value estimated at 765 μg/ml while the IC_50_ values of the extracts ranged from 1.5 to 5 μg/ml. This indicates that the extracts are very potent α-glucosidase inhibitors in comparison with acarbose. Only a few articles had discussed acarbose effect in detail [22-25]. In those studies acarbose was found to exert little inhibition on α-glucosidase activity (IC_50_ ≥ 1000 μg/ml in each case) and authors justified this by the nature of α-glucosidase enzyme which were from mammalian (rat intestine), bacterial (*Bacillus stearothermophilus*), and yeast (*Saccharomyces cerevisiae*) sources. This could justify why all extracts studied here were more active against this enzyme than acarbose. Furthermore, the nature of some extract constituents (phenolics, flavonoids and their glycosides) is in accordance with previous reported works as being effective inhibitors of α-glucosidases [26-29]. For example, water extract of *P. kotschyi* leaves was evaluated by Udeme *et al.* on blood glucose in alloxan-induced diabetic albino wistar rats [3]. To the best of our knowledge, nothing has been reported on *P. senegalensis*’s AGI properties. Here, our results show that both plant species contained active principle(s) which could back up their use in the treatment of type 2 diabetes. It would be interesting to know which molecule or group of molecules are responsible of this activity in each extract. Biological effect-guided fractionation is currently in progress.

The present work also involved the evaluation of the antioxidant activity of these extracts. The two *in vitro* electron transfer methods (DPPH and FRAP) presented consistent results: when the extract showed low IC_50_ value using DPPH test, it exhibited a high FRAP value. Moreover, our investigation on the ability of both extracts to transfer a hydrogen atom showed a high potency with a high ORAC value in both cases. This activity may be due to the presence of phenolic compounds in the extract [30]. Generally, the reducing properties are associated with the presence of compounds which exert their action by breaking the free radical chain through the donation of a hydrogen atom [31]. The ability of these compounds to give hydrogen atoms may be due to their polyphenolic nature. Excepted the antiradical activity of essential oils from *P. kotschyi* root barks, no work on antioxidant activity of alcohol- or water-soluble extract of both plants was previously described [32].

Antioxidants are particularly interesting in drug discovery [33]. Several techniques are used for the determination of antioxidants in plant extracts, however these techniques have some limitations [34,35]. However, *in vitro* tests do not take into account the cells’ physiological conditions, the antioxidant molecules’ bioavailability and the general cellular metabolism. Therefore, in this study, a complementary method was used to evaluate the antioxidant activities of the two extracts directly in living mammalian cells. In the presence of ROS such as hydrogen peroxide (H2O2), lipid hydroperoxides and peroxinitrites, DCFH were oxidized to fluorescent 2^′^,7^′^-dichloro-fluorescein (DCF) [18]. The two extracts showed the capacity to reduce tBuOOH-induced DCFH oxidation in cells, especially *P. senegalensis* extracts. Altogether, our results show that antioxidant activities measured with the cell-based assay are in good agreement with the values obtained using FRAP and ORAC assay. It would be interesting to isolate some polyphenols present in the studied samples to see whether the antioxidant activity on cell-based assay is due to a synergistic effect or not.

Polyphenolic compounds in plants have long been recognized to inhibit the activities of digestive enzymes because of their ability to bind with proteins. Various *in vitro* assays have shown that many plant polyphenols possess carbohydrate hydrolyzing enzyme inhibitory activities [26,28,29]. Since these molecules exert antioxidant effect, it is likely that antioxidant and α-glucosidase inhibitory properties should come from polyphenolic contents.

Phytochemical constituents such as tannins, flavonoids, alkaloids and several other aromatic compounds are secondary metabolites of plants which are used as defense mechanisms against predation by many microorganisms, insects and herbivores [36]. Regarding the absence of data on antibacterial properties, the Minimum Inhibitory Concentration (MIC) method was used to determine the antibacterial activity. Our results suggest moderate effect on Gram-positive bacteria while Gram-negative bacteria were found to be less sensitive to the extracts. A possible explanation for these observations may lie in the significant differences in the outer layers of Gram-negative and Gram-positive bacteria. Gram-negative bacteria possess an outer membrane and a unique periplasmic space not found in Gram-positive bacteria [37]. The resistance of Gram-negative bacteria towards antibacterial substances is related to the hydrophilic surface of their outer membrane which is rich in lipopolysaccharide molecules. These properties constitute a barrier to the penetration of numerous antibiotic molecules. It is also associated with the enzymes in the periplasmic space, which are capable of breaking down the molecules introduced from outside [38]. Gram-positive bacteria do not have such an outer membrane and cell wall structure. Antibacterial substances can easily destroy the bacterial cell wall and cytoplasmic membrane which results in leakage and coagulation of the cytoplasm [39]. The resistance of the Gram-positive *C. difficile* to extracts could be due to its strict anaerobic metabolism, there is no study to justify this yet.

## Conclusion

The results obtained in this study support the use of *P. senegalensis* (leaves) and of *P. kotschyi* (root) as traditional medicinal plants in Benin against some disorders such as hyperglycemia. Our study indicated that these plants possess α-glucosidase inhibitory activity, and potent antioxidant effect. These effects were slightly more pronounced in *P. senegalensis* than in *P. kotshyi* and further investigations of the active natural compound(s) of these plants are in progress. This work is an important step in exploring the pharmacological effect of plants traditionally used in Benin.

## Abbreviations

DW: Dry weight;DPPH: 2, 2-diphenyl-1-picrylhydrazil;FRAP: Ferric reducing/antioxidant power;ORAC: Oxygen Radical Absorbance Capacity;DCFH -DA: Dichlorofluorescin diacetate;MHB: Müller-Hinton Broth;MIC: Minimum Inhibitory Concentration;AGI: α-Glucosidase inhibitors;HAT: Hydrogen atom transfer;ET: Electron transfer;Fe3^+^/2^+^-TPTZ: Ferric/Ferrous tripyridyltriazine

## Competing interests

The authors declare that they have no competing interests.

## Authors’ contributions

FTDB conducted experiments on glucosidase, antioxidant and antibacterial activities, statistical analysis and the draft of the manuscript. ED was responsible for conception and design of the study, statistical analysis and revised the manuscript critically for important intellectual content. CF supervised antibacterial activities. FA participated in design of the study and preparation of the manuscript. DS and JT were revised it critically for important intellectual content. All authors read and approved the final manuscript.

## Pre-publication history

The pre-publication history for this paper can be accessed here:

http://www.biomedcentral.com/1472-6882/13/51/prepub
